# Low Complexity Radar Gesture Recognition Using Synthetic Training Data

**DOI:** 10.3390/s23010308

**Published:** 2022-12-28

**Authors:** Yanhua Zhao, Vladica Sark, Milos Krstic, Eckhard Grass

**Affiliations:** 1 IHP—Leibniz-Institut für Innovative Mikroelektronik, 15236 Frankfurt, Germany; 2 Institute of Computer Science, Humboldt University of Berlin, Rudower Chaussee 25, 12489 Berlin, Germany; 3 Design and Test Methodology, University of Potsdam, August-Bebel-Straße 89, 14482 Potsdam, Germany

**Keywords:** FMCW radar, gesture sensing, machine learning, mmWave, synthetic features

## Abstract

Developments in radio detection and ranging (radar) technology have made hand gesture recognition feasible. In heat map-based gesture recognition, feature images have a large size and require complex neural networks to extract information. Machine learning methods typically require large amounts of data and collecting hand gestures with radar is time- and energy-consuming. Therefore, a low computational complexity algorithm for hand gesture recognition based on a frequency-modulated continuous-wave (FMCW) radar and a synthetic hand gesture feature generator are proposed. In the low computational complexity algorithm, two-dimensional Fast Fourier Transform is implemented on the radar raw data to generate a range-Doppler matrix. After that, background modelling is applied to separate the dynamic object and the static background. Then a bin with the highest magnitude in the range-Doppler matrix is selected to locate the target and obtain its range and velocity. The bins at this location along the dimension of the antenna can be utilised to calculate the angle of the target using Fourier beam steering. In the synthetic generator, the Blender software is used to generate different hand gestures and trajectories and then the range, velocity and angle of targets are extracted directly from the trajectory. The experimental results demonstrate that the average recognition accuracy of the model on the test set can reach 89.13% when the synthetic data are used as the training set and the real data are used as the test set. This indicates that the generation of synthetic data can make a meaningful contribution in the pre-training phase.

## 1. Introduction

Low-cost, miniaturised radars have become increasingly popular in recent years. This has led to a large number of radar-based applications. For example, automotive radar can play an important role in collision avoidance systems. In addition to this, the potential of the radar in the field of medical applications is also being investigated. Applications such as weather radar and ground-penetrating radar reveal a need for such applications and research on radar technology and algorithms is highly desirable.

Traditional human–computer interaction mediums such as buttons, mice and keyboards are not always convenient in certain situations, such as operations in clean rooms. Contactless human–computer interaction requires less touching and is more hygienic. It can also further enhance the user experience. Hand gestures are an important medium for contactless human–computer interaction [1].

The outstanding privacy-protecting character of radar makes it preferable over cameras and its ability to be unaffected by light conditions is again preferable to LIDAR. The frequency-modulated continuous-wave (FMCW) radar is able to detect the distance, velocity and angle of several objects at the same time; hence, it is employed for hand gesture recognition in our work. FMCW radars can suffer from mutual interference. If there are other radars as sources of interferences, the methods in [2] for finding the range, velocity and angle of the target can be referred to. In this paper, only one radar is employed and interference from other radars is not considered.

Hand gesture recognition based on FMCW radar can be grouped into two categories. The first is based on raw data, meaning that the raw data are fed directly into a neural network to classify the hand gestures. The second is based on features, such as range, velocity and angle. These three features are obtained by certain data pre-processing methods and then combined with machine learning methods to achieve hand gesture classification. In this work, feature-based hand gesture recognition will be considered since the size of the radar raw data is large and will increase the training complexity of the neural network.

### 1.1. Related Work of Heat Map-Based Recognition

The features of a hand gesture can be represented in the form of a heat map. Many researchers have made various contributions to the field of heat map-based hand gesture recognition. Heat map-based gesture recognition is a relatively common approach and in [3,4,5,6,7,8,9,10,11] all authors use heat map-based recognition.

In [3], the authors conducted an experiment on gesture sensing using an FMCW radar with a centre frequency of 25 GHz, without considering the recognition of gestures. Later in [4], the authors collected 1714 gesture samples using FMCW radar, which contains ten types of gesture. Then they extracted time-Doppler heat maps from the radar raw data and trained a deep convolutional neural network model. An average recognition rate of 89.1% was achieved. Deep convolutional networks can be very challenging to implement at the hardware level. This is because they have a large number of weights and although it is possible to remove some of the unimportant weights, by pruning and other methods, it does require a lot of computational resources.

In [5], Lien et al. developed a small, low-power radar with a center frequency of 60 GHz. In contrast to [4], a time-Doppler heat map and a time-range heat map were extracted. With a classical random forest classifier, the average recognition accuracy of the four micro-hand gestures was up to 92.10%.

From the raw data of the FMCW radar not only time-distance and time-Doppler heat maps can be extracted, but also a time–angle heat maps. These three types of heat maps are employed as the basis for hand gesture recognition in [9]. The authors adopted background modelling to separate the static background and the moving target so that the feature heat maps were cleaned efficiently. The average recognition rate of six gestures was over 98.93%. The authors employed a pre-trained model, but a large number of weights of the model made implementation on an FPGA problematic.

Similarly, the authors used the multi-stream convolutional neural network (MS CNN) model in [11] to learn features of the dataset for on-air writing recognition. Although the accuracy achieved was very impressive, the drawback, as before, was that the MS CNN would require a lot of hardware resources.

Given the above references, we can state that heat map-based hand gesture recognition can achieve encouraging results, but its disadvantages should not be ignored. Firstly, the process of constructing a heat map is relatively complex and time-consuming. A hand gesture is made up of several frames of data, each of which will form one or more heat maps depending on the combination of features selected. A single hand gesture sample can produce a large number of heat maps, which leads to complex processing. In addition, the hand gesture features in the form of heat maps need to be further fed into a deep convolutional network to extract features, which requires even more computational efforts.

### 1.2. Motivation for Synthetic Data Generation and Related Work

Machine learning-based hand gesture recognition faces data scarcity issues and only a few open radar datasets are available. Many scientists spend a lot of effort and time collecting data. In Google’s Soli project [5], its team collected 5000 samples with 5 participants. For [10], 7200 gesture samples were collected from 20 people. The authors of [6] collected 2750 samples, which involved 11 participants. A total of 1500 and 1200 gesture samples were collected in [9] and [8], respectively.

In [12], the authors utilised a sparse point cloud extraction method and a Message Passing Neural Network (MPNN)-based graphical convolution method for real-time gesture recognition. Despite the reduced computational complexity, all datasets were collected by other teams manually, which involved a lot of time and effort.

Collecting gesture samples with radar is very challenging. It is needed to perform specific hand gestures repetitively, which is time-consuming and labour-intensive. An efficient gesture feature synthesiser would be highly beneficial.

Human motion simulation is a good start for synthetic gestures. Some researchers have already combined human motion simulation with radar sensors. The authors have developed a human walking model in [13]. Afterwards, in [14], this model was employed to construct micro-Doppler spectrograms for gait analysis.

Reference [15] proposed a radar data synthesis process for four hand gestures. The authors used the 3D computer graphics software Blender [16] to build a simple human hand animation that captured the motion trajectories of the hand gestures. The motion trajectories were then utilised to synthesise radar data. The drawback of this work is that only micro-Doppler spectrograms were considered and the model was not tested with real data.

In [17], the authors proposed a radar data synthesis flow for macro-gestures. Seven gestures were simulated as a training set, which was used to train the Multi-Layer Perceptron model and the real data were employed to test the model with an average recognition accuracy of 84.2%. The drawback is that the ranges and angles of the gestures were not taken into account.

A human target model for the flexible simulation of various modalities of gesture was constructed in [18]. It covered the main parts of the body. In a similar way to the work in [17], only the Doppler spectrum was simulated. A CNN model was applied to classify eight macro-gestures with an accuracy of 80.4%.

The authors in [19] converted video footage of human activity into realistic, synthetic Doppler radar data by means of a cross-domain conversion approach to achieve the goal of synthesising radar training data for human activity. Other features such as range and angle were not synthesised.

In view of this, the gesture synthetic training data generator proposed by the authors in [20] can generate range–time heat maps, velocity–time heat maps and angle–time heat maps. Six gestures were synthesised and real data were also employed to test their validity. The authors used the VGG19 [21] pre-trained model to extract the features from heat maps. After that, the XGBoost [22] and Random Forest [23] classifiers were employed to recognise the hand gestures. The achieved average accuracy was 84.93% and 87.53%, respectively.

### 1.3. Contributions

To reduce the complexity of processing and tackle data scarcity, the main contributions of this paper are as follows.

- A simplified gesture recognition algorithm is proposed. The features of the gestures are represented as one-dimensional vectors instead of images.
- A simplified synthetic hand gesture feature generator is presented. As the hand gesture features extracted from the real data are simplified to one dimension, the synthesis processes from [15,17,18,19,20] are no longer needed. In our simplified synthetic hand gesture feature generator, the generation of radar raw data is skipped.
- The impact of range, velocity and angle features, extracted from a real data set, on the accuracy of gesture recognition is analysed. The experimental results reveal that all three features have a positive effect on gesture recognition. For the evaluation scenarios with a single feature, the average recognition rate based on the velocity feature alone achieves the highest recognition rate on the test set, with a support vector machine (SVM) classifier, which is 87.59%. For the different feature combination evaluation scenarios, the average recognition rate based on the three features yields the best result with an average recognition rate of 98.48%.
- The impacts of the synthetic data set on the recognition accuracy of the real gesture data set are investigated. The SVM classifier trained with the synthetic data has an average recognition accuracy of 89.13% on the real data.

The remainder of the paper is organised as follows: Section 2 describes the FMCW radar system. Section 3 introduces low computational complexity algorithms for extracting features from radar raw data and Section 4 presents a synthetic hand gesture feature generator. Section 5 presents the experiments and results. The conclusions are given in Section 6.

## 2. FMCW Radar System

**Figure 1:**
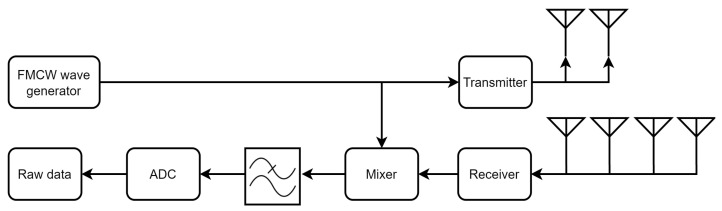
FMCW radar system.

The system architecture of the FMCW radar is illustrated in Figure 1.

**Figure 2:**
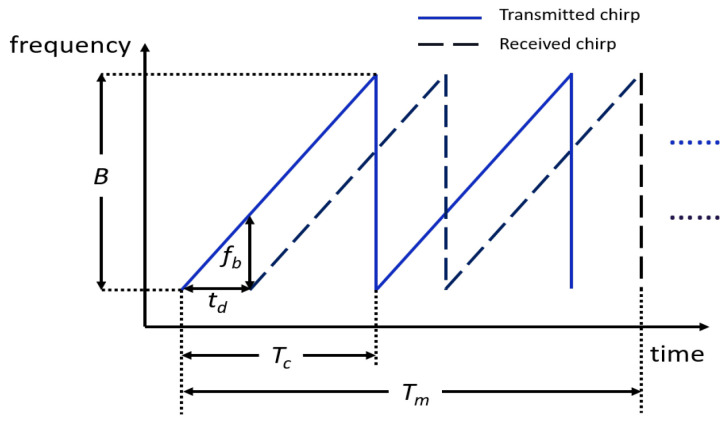
FMCW waveform.

The classical waveforms of FMCW radar are rectangular, upward sawtooth, triangular and staircase voltage waves. Since the hand gesture speed is not as high as an aircraft’s, the upward sawtooth waveform is used in our radar. Firstly, the waveform generator generates an upward sawtooth wave as depicted in Figure 2. The bandwidth of the waveform is *B*. The solid blue line represents the transmitted wave and the dashed black line is the received wave. The frequency slope of the waveform is:

(1) $$s=\frac{B}{T_{c}}.$$

The signal is emitted into space by the transmitter via the transmitting antennas. Our radar has two transmitting and four receiving antennas. The distance between the transmitting antennas is two wavelengths and the distance between the receiving antennas is half a wavelength. A transmitted wave can also be referred to as a chirp. The equation of the transmit chirp is given in (2):

(2) $$T_{x}\left(t\right)=A\exp\left(j\left(2\pi f_{c}t+\pi st^{2}\right)\right).$$

where the amplitude of the chirp is denoted as *A* and the starting frequency is represented by $f_{c}$. $T_{c}$ is the duration of the chirp.

When a wave encounters any object in space, it will be reflected back to the receiver. The object could be our target or any other irrelevant object. The distance between the target and the radar is assumed as $r_{0}$, then the time delay $t_{d}$ between the received and transmitted waves can be represented as:

(3) $$t_{d}=\frac{2r_{0}}{c}.$$

where *c* is the velocity of light.

As the waveform loses energy as it travels through the air and is bounced by objects, there is an amplitude attenuation μ and phase shift in the received waveform. The received wave is defined as follows:

(4) $$R_{x}\left(t\right)=\mu A\exp\left(j\left(2\pi f_{c}\left(t-\frac{2r_{0}}{c}\right)+\pi s{\left(t-\frac{2r_{0}}{c}\right)}^{2}\right)\right).$$

In the next step, the received and transmitted waves will be mixed in a mixer and passed through a low-pass filter to remove the high-frequency components and preserve the low-frequency signal. The remaining signal at this stage is known as the intermediate frequency signal or beat signal. As shown in Figure 2, the slope and time delay of the waveform are *s* and $t_{d}$, respectively, and the frequency of that beat signal can then be given as $f_{b}$:

(5) $$f_{b}=st_{d}=\frac{2Br_{0}}{cT_{c}}.$$

The beat signal can be defined as follows:

(6) $$\begin{array}{cc} B(t) & =\mu A^{2}\exp\left(j\left(2\pi f_{c}\frac{2r_{0}}{c}+\pi s\frac{4r_{0}}{c}t-\pi s{\left(\frac{2r_{0}}{c}\right)}^{2}\right)\right)\\ \; & =\mu A^{2}\exp j(\pi s\frac{4r_{0}}{c}t+2\pi f_{c}\frac{2r_{0}}{c}-\pi s{\left(\frac{2r_{0}}{c}\right)}^{2})\\ \; & =\mu A^{2}\exp\left(j\left(2\pi st_{d}t+\underset{\phi (t)}{\underset{︸}{2\pi f_{c}\frac{2r_{0}}{c}-\pi s{\left(\frac{2r_{0}}{c}\right)}^{2}}}\right)\right)\\ \; & =\mu A^{2}\exp\left(j\left(2\pi f_{b}t+\phi \left(t\right)\right)\right)\\ \; & =\mu A^{2}\exp\left(j\left(\frac{4\pi Br_{0}}{cT_{c}}t+\phi \left(t\right)\right)\right). \end{array}$$

In the final step, the beat signal is digitalised by an analogue-to-digital converter (ADC).

## 3. Feature Extraction from Radar Raw Data

In this section, the process of extracting gesture features from the radar raw data is presented.

### 3.1. Range and Velocity Extraction

**Figure 3:**
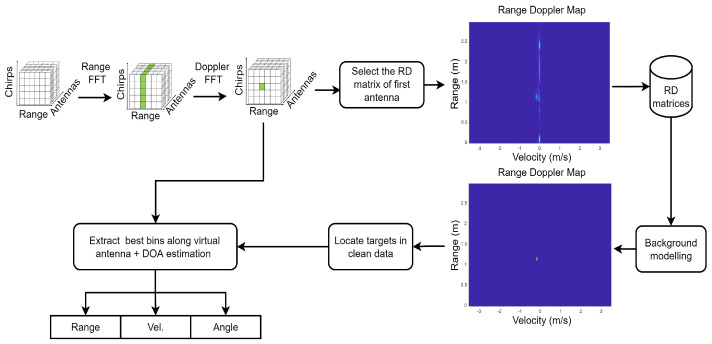
Processing chain for feature extraction from radar raw data.

As illustrated in Figure 3, the structure of a frame of radar raw data has three dimensions, namely range, chirps and antennas. There are eight virtual antenna channels in one frame of the data. Each antenna channel has M chirps and each chirp has N range bins.

To derive range and velocity information, a Fast Fourier Transform (FFT) is applied to the range and chirp dimensions. As depicted in Figure 3, after the range FFT, the range information of the target is highlighted. Then after the Doppler FFT, the range-Doppler (RD) matrix is formulated and the velocity information of the target is enhanced.

FMCW radar detects not only dynamic objects but also static ones. The radar data contain static clutter and static backgrounds, which can be disruptive to gesture recognition. Therefore, it is mandatory to take measures to combat irrelevant noise and static background.

Background modelling based on the Greedy bilateral smoothing (GreBsmo) [24] is employed to remove static clutter and static background objects, while the dynamic objects are retained. The data *X* can be decomposed to the background *L*, clean data *S* and noise *G*.

(7) X=L+S+G.

After obtaining the RD matrices, the RD matrix for the first antenna channel is selected and saved in the data container. After executing the data for a whole hand gesture, the background modelling is carried out. The two heat maps in Figure 3 indicate the contribution of background modelling. After background modelling, the RD heat map becomes clean and only the target object remains. Thus the range and velocity of the target can be located more accurately in the RD matrix. It is assumed that the moving target in the data set has only one hand and is the main component. The index of that best bin can be derived by finding the maximum value in the RD matrix. Once the index is found, it is possible to calculate the range and velocity of the target.

### 3.2. Angle Extraction

The target bin is defined as the index of the RD matrix where the target is located. The best bin is searched along the antenna index. The bins being extracted are a 1 × 8 vector, denoted by e.

By performing Fourier beam steering (FB) [25,26] on e, angle information is derived. The virtual beam steering matrix is represented as:

(8) $$V\left(\Theta ,q\right)=\exp\left(j2\pi \left(-\frac{Q-1}{2}+q\right)\frac{\Delta d}{\lambda }\sin(\frac{\pi }{180}\Theta )\right).$$

where *Q* is the total number of virtual antennas, q∈{1,2,3,…,8}. λ stands for the wavelength. Δd is the spacing distance between the receiving antennas and its value is $\frac{1}{2}\lambda$. The scanning scope of the angle Θ is [−90,90] and the step size is 1.

(9) $$I=V\cdot e^{T}.$$

**Figure 4:**
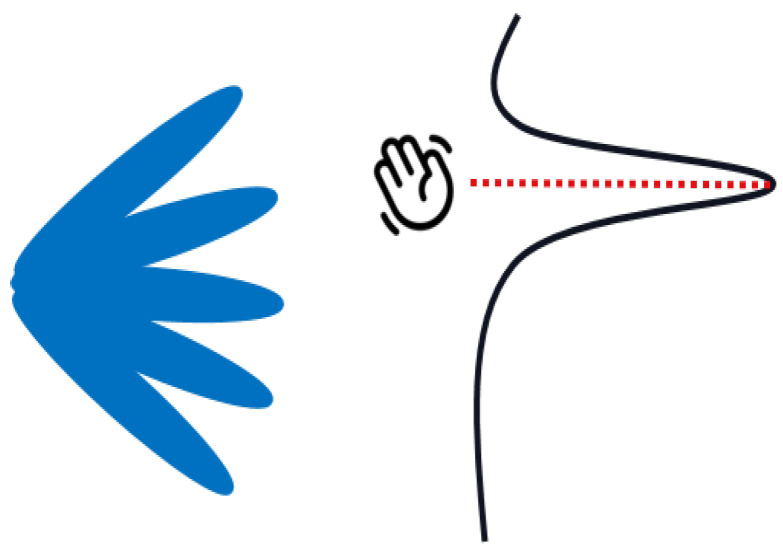
Fourier beam steering.

It is assumed that there is only one target and therefore the angle corresponds to the maximum value in *I*, as depicted in Figure 4.

The processing sequence of our approach makes the angle estimation more stable and accurate. By selecting the best bins in the RD matrices, the angle information of irrelevant objects will be excluded. This is exactly the opposite of the processing order of unmanned aerial vehicle (UAV) swarms detection using the radar in [27,28]. In contrast to UAV swarm detection, only one target is taken into account in gesture recognition and a linear array of antennas is used in the radar.

**Figure 5:**
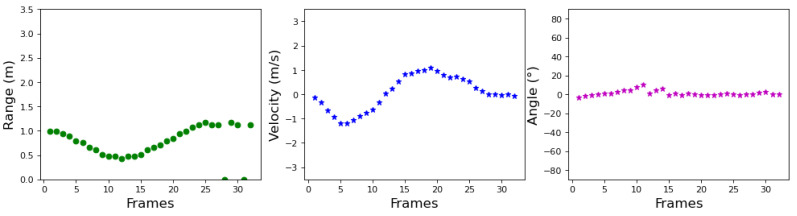
An example of gesture “push pull”.

In contrast to heat map-based gesture recognition, a gesture sample has 32 frames only, leading to a 1 × 96 feature vector. The features of a sample are shown in Figure 5. The overall process of extracting features from the radar raw data is shown in Algorithm 1.
**Algorithm 1:** Feature extraction from radar raw data.
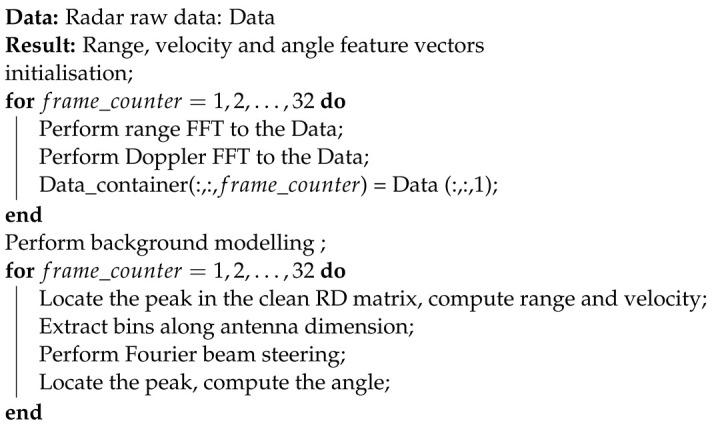



## 4. Synthetic Feature Generator

**Figure 6:**
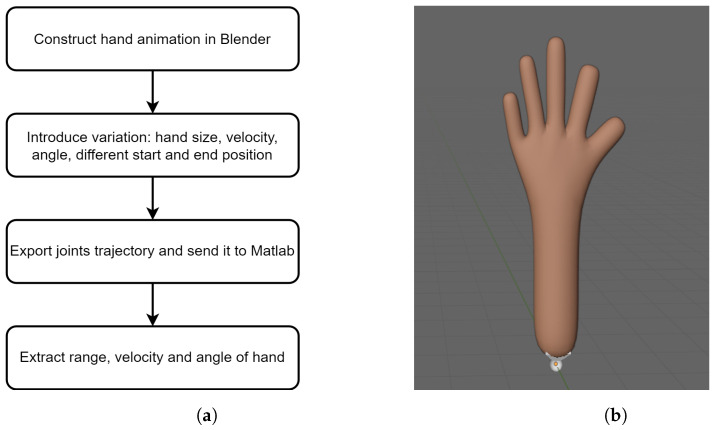
(**a**) The workflow of the gesture feature synthesiser; (**b**) an example of hand animation.

To match the gesture features with the real data, a synthetic generator of hand gestures is adapted. In this section, the workflow of the gesture feature synthesiser is proposed as illustrated in Figure 6a.

### 4.1. Generator Architecture

Our work uses Blender to animate hands. Blender provides the armature API and Python scripting. Python scripts make it easier to produce a large number of hand gestures. In Blender, the skeletal structure and joints of the hand are constructed, as depicted in Figure 6b.

Human hands vary in size and have different habits of movement. Therefore, the simulator can reproduce different joint types and the hand can be simulated with varying velocities, angles and start–stop positions. The trajectories are fed into Matlab to calculate the features of the hand movement, namely range, velocity and angle.

### 4.2. Feature Extraction

**Figure 7:**
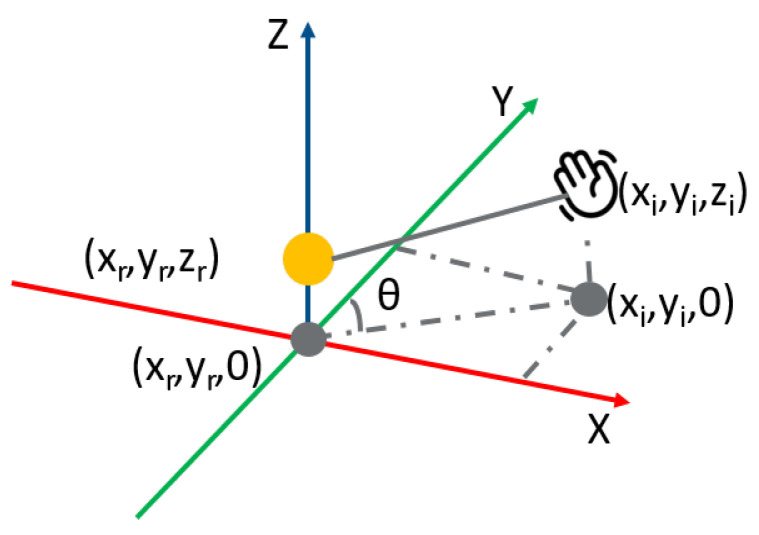
Target and radar in the coordinate system.

The process of extracting features from a trajectory is illustrated in Algorithm 2. Figure 7 displays the radar and hand in the 3D space. The orange dot represents the radar with the location represented as $\left(x_{r},y_{r},z_{r}\right)$. The position of one joint is denoted as $\left(x_{i},y_{i},z_{i}\right)$. The distance between these two points in space is calculated by (10):

(10) $$d=\sqrt{{\left(x_{i}-x_{r}\right)}^{2}+{\left(y_{i}-y_{r}\right)}^{2}+{\left(z_{i}-z_{r}\right)}^{2}}.$$

The distance from each joint of the simulated hand to the radar is calculated according to (10) and averaged as the distance between the hand and the radar for one frame.

For velocity, the initial value of velocity is set to 0. The formula for the calculation is given in (11). $d_{k}$ is the distance between the radar and the hand in the current frame. $d_{k-1}$ is the distance in the previous frame while $T_{m}$ represents the duration of a frame. The difference between the distances of adjacent frames divided by the frame duration gives the hand velocity in the current frame.

(11) $$v=\frac{d_{k}-d_{k-1}}{T_{m}},k\in \left\{2,3,\ldots ,32\right\}.$$

**Algorithm 2:** Synthetic feature extraction.
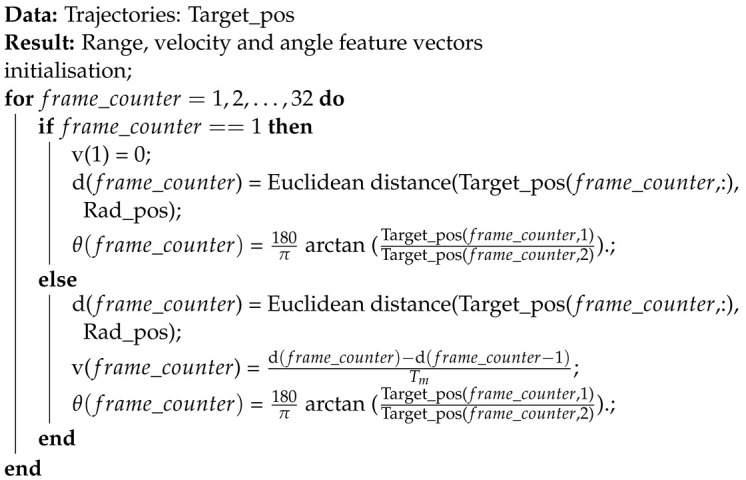


Our radar can only detect the azimuth of the object. In Figure 7, the radar and the hand are projected onto the same plane. The grey dot is the projection of the hand. The projection of the radar is then at the origin of the coordinate system and the azimuth angle θ between radar and a joint is derived by (12). The angle between the hand and the radar is averaged over all joints of the hand.

(12) $$\theta =\frac{180}{\pi }\arctan\left(\frac{x_{i}}{y_{i}}\right).$$

To make the synthesised hand gesture features more realistic, random noise is added to the extracted features.

### 4.3. Recognition Pipeline

The features of the hand gestures are fed directly into the support vector machine (SVM) [29] after they have been extracted based on the approach described previously. The support vector machine algorithm is particularly efficient in terms of memory and it performs better if there is a significant margin of separation between hand gestures.

## 5. Experiment and Evaluation

### 5.1. Radar Settings

The radar used for the experiments is a Texas Instruments (TI) AWR1642 [30], which operates at a starting frequency of 77 GHz and a maximum bandwidth of 4 GHz. It has two transmitting and four receiving antennas. The specific parameters used for the experiments are listed in Table 1. This radar also needs a raw data acquisition board. For this reason, we use TI DCA1000EVM [31].

### 5.2. Dataset

**Figure 8:**
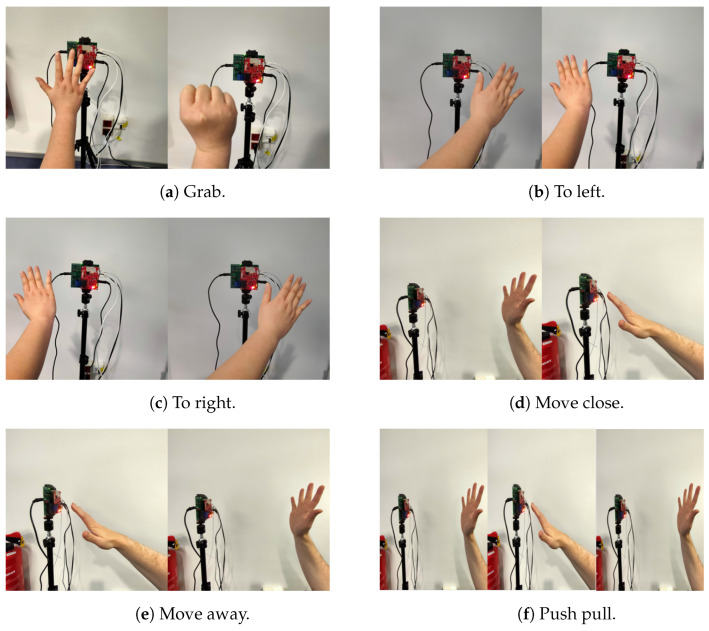
Hand gesture type.

Two datasets are collected in this study, a real dataset based on AWR1642 and a synthetic dataset synthesised by the gesture feature simulator. There are six hand gestures in the dataset as shown in Figure 8: “grab”, “to left”, “to right”, “move close”, “move away” and “push pull”. These are gestures that are commonly used in daily life. The real data set contains 250 samples of each gesture gathered from two participants in an indoor environment. Our low computational complexity approach takes an average of 0.0157 s to extract features from the raw data of each sample. The synthetic data set has 2700 samples per gesture.

### 5.3. Feature Comparison

Next, features from the real dataset and synthetic features are analysed and compared. The features of the hand gestures are illustrated in a 3D scatter plot. For readability, only five samples of each gesture are displayed. A gradient colour has been used for the feature scatter plot, from dark to light. The starting frame of the feature for each sample is the darkest colour and the last frame is the lightest colour.

**Figure 9:**
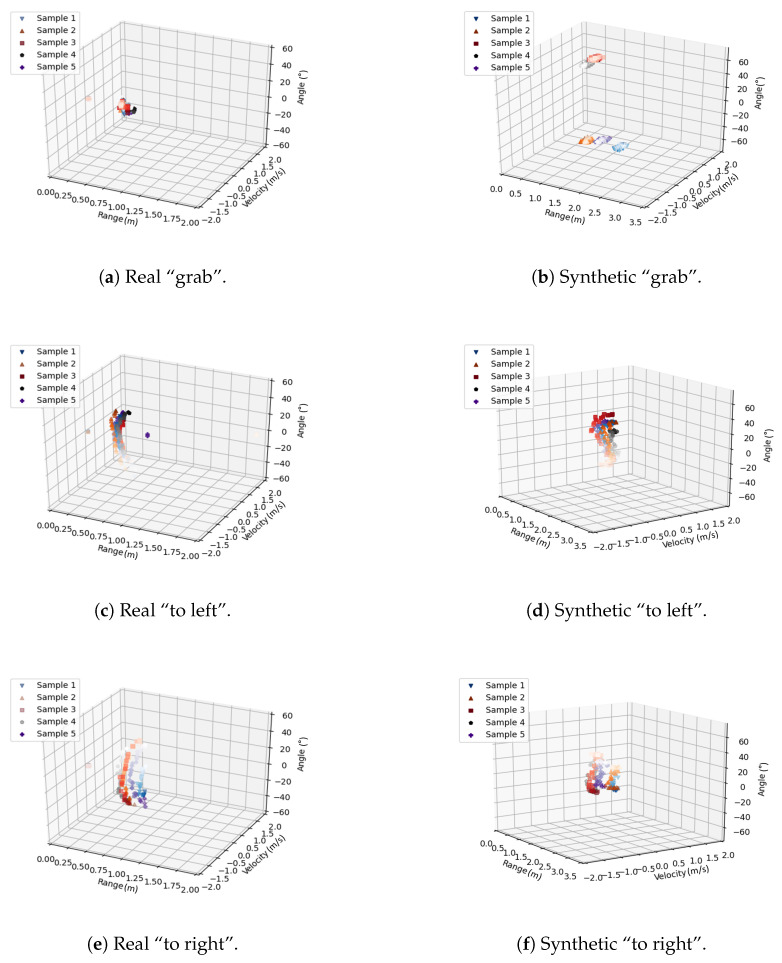
Comparison of “grab”, “to left” and “to right”.

Figure 9a,b indicate the features of the real “grab” and the synthetic “grab”. This gesture does not change much in terms of range, velocity and angle and that is because the grabbing, with the fingers slowly closing together, does not change much in motion.

“To left” gesture is compared in Figure 9c,d. The real “to left” and the synthetic “to left” both have a drastic change in the dimension of the angle. A trend from larger to smaller angles can be seen on both figures. The hand moves from right to left and there will surely be a changing of angle relative to the radar. In contrast, there is not a lot of variation but a small decreasing and increasing trend in the range. This comes from the fact that the midpoint of the gesture is closer to the radar than the start and end points. Some small variation is expected because human movements are not perfectly aligned with the radar. The same is true for the velocity because only movements towards or away from the radar will influence the measured velocity.

The change in angle features for “to right” is the opposite of “to left” in Figure 9e,f. The real “to right” angle changes from a negative to a positive value. The other features of the gesture are identical to the “to left” gesture.

**Figure 10:**
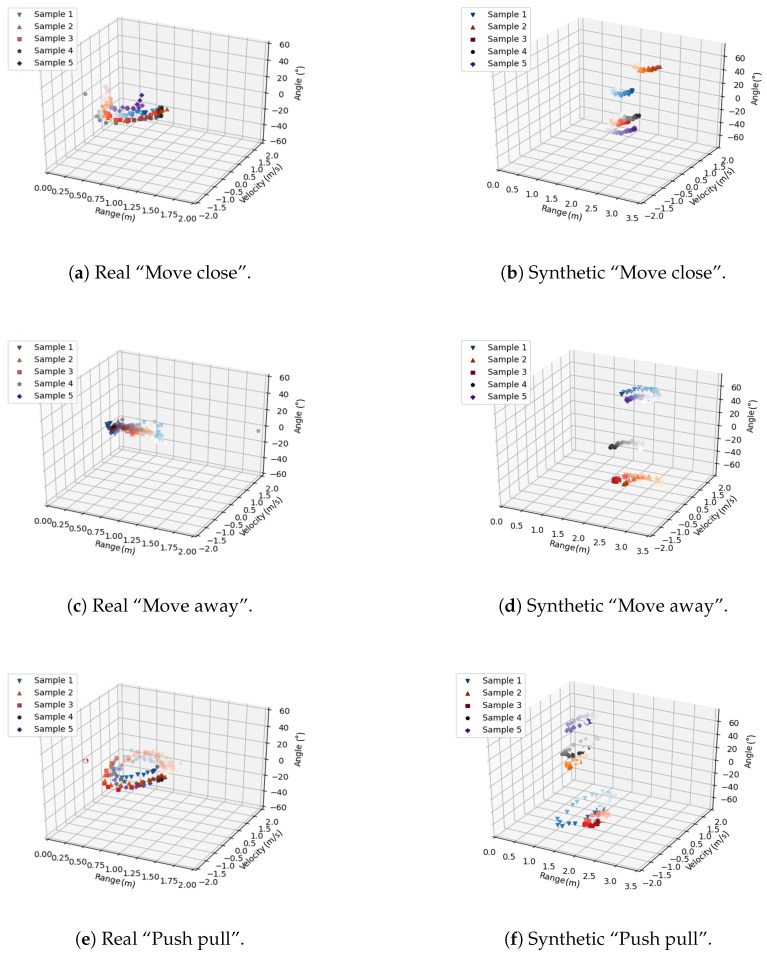
Comparison of “move close”, “move away” and “push pull”.

Figure 10a,b present the 3D features of the real “move close” and the synthetic “move close”. The characteristic element of this gesture is the change of the range from larger to smaller values. This is because the hand is gradually moving closer to the radar. The velocity changes from zero to a negative value and then back to zero when the movement is finished. There is almost no change in angle. Some variation can be observed because the target is not perfectly positioned to move directly towards the radar.

For the “move away” gesture, the range between the hand and radar gradually increases as the hand moves in Figure 10c,d. In addition, the trend of the velocity is the opposite of that of “move close”. The angle does not have much variation and the little variation observed behaves in the opposite manner of “move close”.

“Push pull” is a combination of “move close” and “move away”. The hand first approaches the radar and then moves away. The distance decreases and then increases. The corresponding velocity changes towards positive and negative values. The angle does not change obviously. The feature of “push pull” in Figure 10e,f is in the form of a closed circle. There is a high similarity between the real and synthetic features.

### 5.4. Feature Distribution

**Figure 11:**
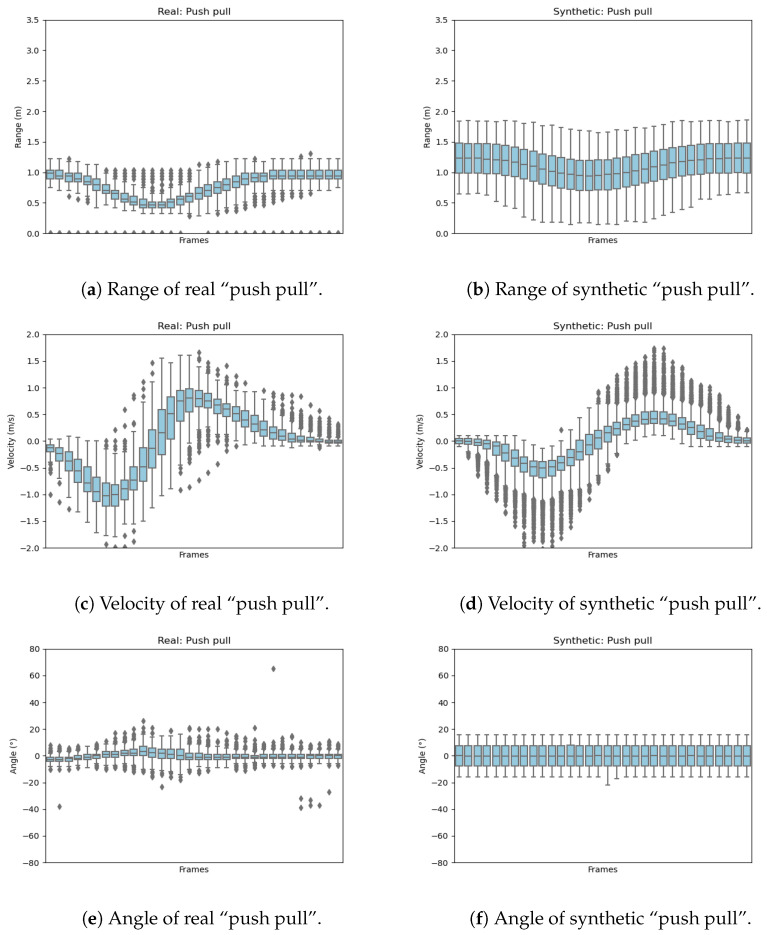
Box-plot for feature distribution of real and synthetic “push pull”.

To show the distribution of features for one gesture from the entire data set, the distribution of features for the gesture “push pull” is illustrated in Figure 11.

This “push pull” distribution is based on 250 samples in the real data set and 2700 samples in the synthetic data set. It can be concluded from Figure 11a,b that the distribution zone of the range features for most of the “push pull” samples first decreases and then increases over time. The distribution of synthetic range features is relatively wider; this is due to the richness of the hand start and end position variations during the simulation of the trajectory. The synthetic data are purposefully created with as much variation as possible while still performing the hand gesture. The goal of this is to represent as many different ways to perform this gesture as possible. The speed of the real gestures seems to be higher than most of the synthetic data but the velocity of the real gesture is still included in the synthetic data. The velocity pattern for this gesture can be seen in both data sets. For the angle, the synthetic data cover a wider range of values; this will lead to a better classification performance if the gesture is performed from different angles. The overall pattern is similar to the real data. It can be seen that the real gesture was usually measured directly from the front. The synthetic data also cover the gesture if it is performed at an angle.

### 5.5. Feature Impact Analysis

From the radar raw data, three features have been extracted, namely range, velocity and angle. In this subsection, the effect of feature combinations on the accuracy of gesture recognition will be analysed. To analyse the impact of one type of feature, a data set containing a single feature is fed into the SVM for training and testing. A total of 50% of the real data set was randomly selected as the training set and the other 50% as the test set.

**Figure 12:**
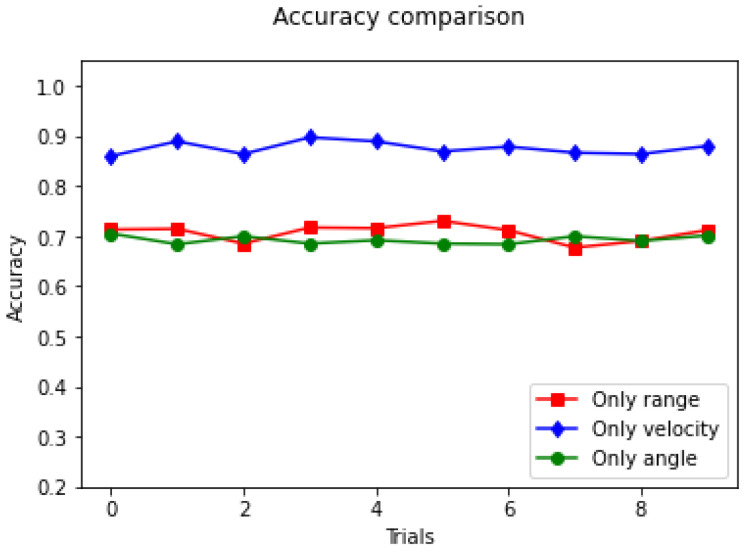
Accuracies based on single feature.

The experiment is repeated ten times and the recognition rates for the test set are summarised in Figure 12 and Table 2. As can be seen in the figure, velocity plays a significant role. For velocity alone, the average recognition accuracy in the test set was as high as 87.59%.

**Figure 13:**
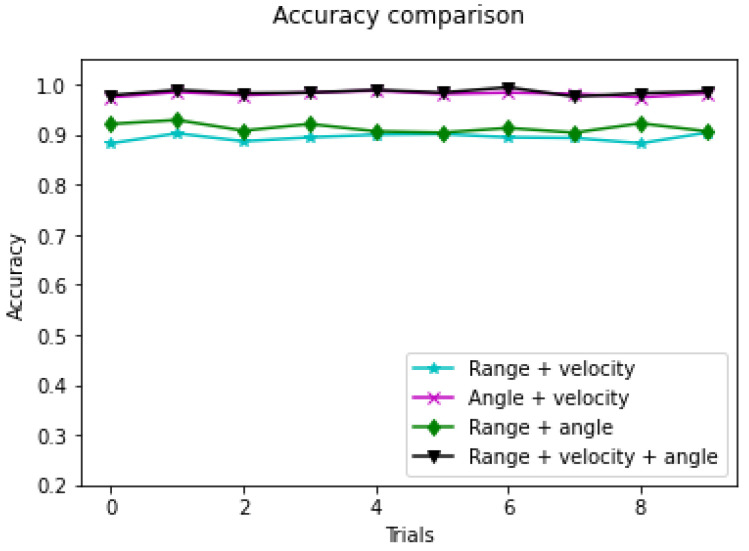
Accuracies based on multi features.

Furthermore, the combination of features is evaluated. The results based on the combination of different features are illustrated in Figure 13 and Table 3. Recognition rates based on multiple features are higher compared to a single feature. The average accuracy based on the three features is as high as 98.48%. The combination of velocity and angle can achieve accuracies of up to 98.15% on the test set.

From the outcomes, it can be derived that the velocity is more recognisable among the three features. However, based on velocity alone, the recognition rate of gestures is below 90%. When range and angle are also taken into account, the recognition accuracy on the test set is improved considerably.

### 5.6. Synthetic Feature Impact Analysis

Three evaluation scenarios are defined. Scenario I is a random selection of 2% of the real data as the training set and the rest of the real data are used as the test set. Scenario II uses the synthetic data set as the training set and the entire real data set as the test set. Scenario III takes 2% of the real data and the entire synthetic data set as the training set and the remaining 98% of the real data set as the test set.

**Figure 14:**
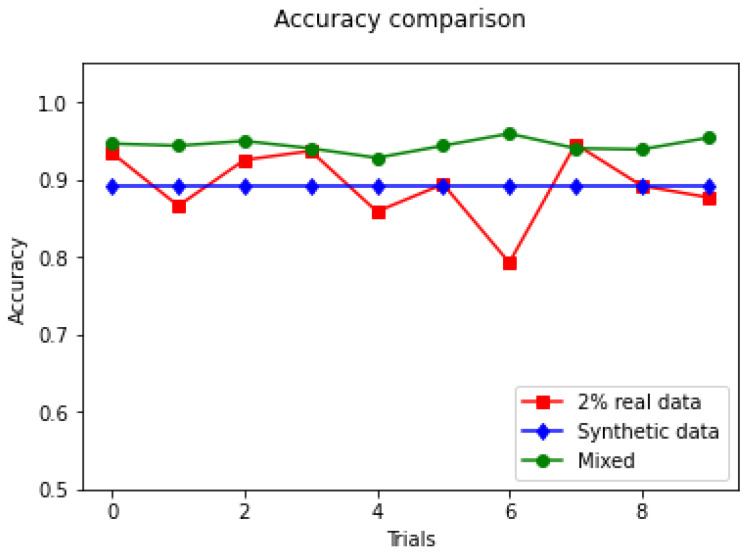
Accuracy of test set.

The recognition accuracy of the SVM on the test set is given in Figure 14 and Table 4. The experiment is repeated ten times.

The red line represents the accuracy of scenario I on the test set. It can be seen that the high fluctuation is due to a low amount of real data and the quality of the selected data varies. If the 2% randomly selected real samples from the real data set cover a large variation and have a high quality, then it is likely to yield good results on the test set. If the randomly selected samples have low quality or little variation, there is a chance that worse results can be obtained on the test set. The results for scenario II are indicated by the blue line. The blue line is stable because the test set and training set have not changed. The green line represents the result of scenario III. These results are the best among the scenarios. This shows that the synthetic data are able to enhance data sets of real data to achieve a performance that is higher than the individual data sets.

The average accuracy of ten attempts for the three scenarios is given in Table 4. When the SVM classifier uses only the synthetic data set as the training set, the model has an average accuracy of 89.13% on the test set. When the synthetic data set is combined with a small amount of real data, better performance can be obtained, with an average recognition rate of 94.43%.

One more advantage of synthetic data is that they cover a wider range of possible gestures. The training and test sets are very similar, so a small amount of real data can lead to high performance in the test set. A test set that differs in these features but still includes valid gestures may result in poorer performance for a model trained on real data. Synthetic data has a wider range of feature values, so it is able to generalise better and cover different test sets in a better way.

### 5.7. Comparison with Other Works on Synthetic Feature Generators

In Table 5, a comparison with other works is summarised. Previous work such as [17] combined animation modelling in Blender with a simulated FMCW radar sensor to simulate velocity–time heatmap features for seven types of macro-gesture. In [18], the human model was constructed and Doppler spectrograms for eight macro-gestures were simulated. The authors synthesised six activities in [19] by transforming the data from the camera into Doppler heat maps. The number of samples in their test set is 720.

Reference [20] simulated range–time, velocity–time and angle–time heatmap features for six types of hand gestures by combining gestures constructed in Blender and a simulated FMCW radar. For both, the classifiers were trained with synthetic data sets and the models were tested with real data sets. However, this does not suit our features extracted from the radar raw data.

The features of our sample are saved in a csv [32] file. In Table 5, it can be observed that the features extracted from a sample of [20] are 8 to 12 times larger than the features extracted by our new approach. Compared to [17,18,19,20], the average recognition accuracy on the real data set is higher, which indicates the strength of this work. In addition to this, our synthetic feature generator does not contain radar signal simulation, which reduces the computational effort significantly. More importantly, the dataset extracted by our method does not need to be fed further into the machine learning algorithm for feature extraction. This will help to save hardware resources significantly.

## 6. Conclusions and Future Work

In this work, a low computational complexity hand gesture feature extraction methodology based on FMCW radar and a synthetic hand gesture feature generator are proposed.

The impact of range, velocity and angle on the accuracy of gesture recognition is analysed. The combination of the three features leads to the highest accuracy.

Compared to other works, our synthetic gesture feature process avoids the generation of radar raw data and is more straightforward. In contrast to heat map-based recognition, additional feature extraction can be skipped in the training phase.

The synthetic data set is used to train a SVM classifier to recognise six different hand gestures in the real data set, which are acquired with an AWR1642 FMCW radar. The algorithm achieves an average recognition accuracy of 89.13% on the real data set. By combining a small amount of the real data set with the synthetic data set, an average recognition accuracy of 94.43% is obtained on the real data set. Thus, we demonstrate the effectiveness of our raw data pre-processing approach and feature synthetisation process.

In the future, more gesture types, different frequency bands and cross-platform gesture recognition should be investigated. We would like to verify the performance of our selected models in different aspects in future work, for example, by adding more datasets with different characteristics.

## Figures and Tables

**Table 1:** Radar parameters.

Parameter	Value
Starting frequency	77 GHz
Transmitting antennas	2
Receiving antennas	4
Number of range bins	64
Number of chirps per frame	255
Bandwidth	3.8 GHz
Chirp duration	38 μs
Frequency slope	100 MHz/μs
Frame duration	71 ms
Number of frames per gesture	32

**Table 2:** Comparison of accuracy based on a single feature.

Feature Type	Average Accuracy	Standard Deviation
Only range	70.69%	1.59%
Only velocity	87.59%	1.23%
Only Angle	69.28%	0.78%

**Table 3:** Comparison of accuracy based on feature combination.

Feature Combination	Average Accuracy	Standard Deviation
Range + velocity	89.43%	0.76%
Velocity + angle	98.15%	0.42%
Range + angle	91.37%	0.87%
Range + velocity + angle	98.48%	0.52%

**Table 4:** Comparison of accuracy.

Scenarios	Average Accuracy	Standard Deviation
Scenario I: 2% real data	89.21%	4.43%
Scenario II: Synthetic data	89.13%	0.00%
Scenario III: Mixed	94.43%	0.82%

**Table 5:** Comparison with other works.

Ref.	Sample Size	Real Samples	Testset Average Accuracy
[17]	**-**	1050	84.2%
[18]	**-**	3354	80.4%
[19]	**-**	720	81.4%
[20]	8–12 KB	1500	87.53%
This work	1 KB	1500	89.13%

## Data Availability

Not applicable.
